# APOC1‐Associated VLDL‐Pathway Dysregulation in Papillary Thyroid Carcinoma: A Reproducible Cross‐Cohort Transcriptomic Signature Associated With the Diagnostic‐to‐Treatment Interval

**DOI:** 10.1111/cbdd.70403

**Published:** 2026-09-15

**Authors:** Yinfeng Li, Ge Meng, Min Peng, Guangyan Chen, Yin‐Ping Zhang

**Affiliations:** ^1^ Faculty of Nursing Xi'an Jiaotong University Health Science Center Xi'an Shaanxi P.R. China; ^2^ The Second Affiliated Hospital of Xi'an Jiaotong University Xi'an Shaanxi P.R. China

**Keywords:** apolipoprotein C1, diagnostic‐to‐treatment interval, Mendelian randomization, papillary thyroid carcinoma, preoperative risk stratification, VLDL metabolism

## Abstract

The diagnostic‐to‐treatment interval (DTI) of papillary thyroid carcinoma (PTC) has long been regarded as an inactive administrative period. We expected to find that DTI is a window of high metabolic activity where apolipoprotein dysregulation may lead to harm. A four‐phase integrated design was used. Phase 1 analysed transcriptomic and clinical data from TCGA‐THCA (*n* = 209) to find DTI‐associated molecular alterations, and used a maximally selected rank statistic to set the optimal time window. Phase 2 verified the lipoprotein signature in three independent GEO cohorts (GSE29265/GSE33630/GSE60542). Phase 3 used XGBoost regression in conjunction with SHAP (SHapley Additive exPlanations) to explore gene–gene interactions and predictive structures. Phase 4 used two‐sample Mendelian randomization to determine whether genetically determined levels of the 233 metabolic biomarkers were associated with PTC. A 77‐day threshold optimally stratified postoperative complete response rates (91.5% for DTI ≤ 77 days vs. 79.0% for DTI > 77 days; *p* = 0.035). Differential expression analysis found 77 genes that were significantly associated with prolonged DTI, and the enriched pathways included PPAR signalling, fat digestion and apolipoprotein particle dynamics. Linear regression showed that longer DTI was associated with higher expression of APOA1 and APOC1. XGBoost–SHAP analysis showed that APOA1 and APOC1 were the top predictive features, and their interaction explained most of the non‐additive variance. Cross‐cohort validation showed that APOC1 and LPL were consistently upregulated and VLDLR was downregulated in all GEO datasets. Disease‐state predictive model building found that the LPL–APOC1 axis effectively distinguished tumour from normal tissue. In two‐sample MR analysis of 233 metabolic traits, genetically predicted VLDL and triglyceride‐rich lipoprotein traits showed a predominantly risk‐increasing direction of effect, and HDL cholesterol exhibited a protective effect, which was consistent with the transcriptomic results; however, no individual exposure remained significant after multiple testing correction in either outcome dataset, so the genetic analysis provided support for and extended the hypothesis rather than confirming it. Based on the above analysis, the pre‐operative period of PTC may be metabolically active rather than quiescent; however, this has not been confirmed in future studies. Dysregulation of the APOC1‐associated VLDL pathway is a reproducible cross‐cohort transcriptomic signature in PTC; although genetic analysis has been conducted directionally, strong causal evidence has not been obtained. Therefore, at the same time, we will shift from time‐limited to metabolically targeted preoperative risk assessment.

## Graphical abstract

Across four independent thyroid cancer cohorts, multi‐omics analysis identifies a reproducible APOC1–LPL–VLDLR lipid‐pathway signature linked to the preoperative diagnostic‐to‐treatment interval, nominating triglyceride‐rich lipoprotein metabolism as a candidate window for monitoring and a potential metabolic axis in papillary thyroid carcinoma.

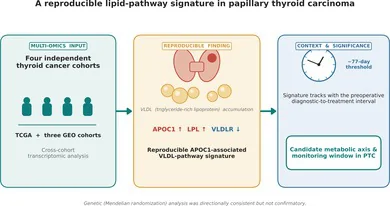

## 1 Introduction

Typical examples of endocrine tumours are papillary thyroid carcinomas (PTCs). With the spread of high‐resolution ultrasound and fine‐needle aspiration biopsy, many slow‐growing microcytic tumours of the thyroid (PTC) have been diagnosed (Bray et al. 2024; Zheng et al. 2024). As a result of this change in epidemiology, active surveillance (AS) for low‐risk lesions has been introduced at institutions to believe that a delay in treatment will not harm the prognosis of papillary thyroid cancer (PTC) (Lopez et al. 2023; Frosch et al. 2023; Zhang et al. 2026). Patients selected for AS are followed up over time and not immediately operated on (Ito et al. 2024; Walter et al. 2023). However, the idea of ‘treatment delay’ has implicitly expanded beyond organised AS, and intentional observation can also lead to fragmented care paths that result in involuntary waiting. For patients not in the AS protocol, the extended time from diagnosis to treatment is generally due to systemic healthcare inefficiencies and not delayed informed clinical choices (Liu et al. 2024; Li, Cao, and Jiang 2025). The preceding assumption of clinical stability for PTC in the DTI is required to guarantee the safety of AS, but it contradicts the basic idea of cancer biology that tumour populations evolve through ecological selection (Zhang et al. 2026). Therefore, the outwardly passive waiting period may be an active window of microenvironmental adaptation, and the molecular effects of this adaptation have not yet been studied.

Molecular landscape studies of PTC have been carried out on a large scale to identify mutually exclusive driver mutations, such as BRAF V600E, RAS family mutations and rearrangements involving RET/PTC or NTRK in most cases (Mohammed et al. 2026; Prete et al. 2020). Although genetic profiling has achieved some classification and targeted‐therapy development in recent years, little has been done to study how these changes occur in living tumours prior to surgery. Assuming that a genomically defined tumour has a fixed phenotypic path from diagnosis to surgery ignores changes in transcription and metabolism that may have occurred during this time. It is unknown whether the delay in treatment leads to other modifications of the molecular environment of metabolism and stress‐response pathways; therefore, it cannot be determined whether such factors affect the efficacy of treatment or perioperative outcomes without affecting survival (Hanahan 2022; Tufail et al. 2024). Although some cohorts have not found an association between surgical delay and all‐cause mortality, analysis of the National Cancer Database (NCDB) and SEER‐Medicare data shows that a delay of more than 180 days increases thyroid cancer‐specific mortality by about four times compared with intervention within 90 days (Fligor et al. 2021; Davies and Welch 2010; Chaves et al. 2023). This difference is probably due to varying definitions of delay, different endpoints and different comorbidities among the patients, not random errors. Epidemiological data have not shown a simple ‘time‐toxicity’ phenomenon; rather, changes have occurred at the molecular level over time (Chaves et al. 2023; Quail and Joyce 2013; Junttila and de Sauvage 2013). DTI may enhance adaptation by altering the regulatory mechanisms of immune editing, stromal remodelling and metabolic reprogramming.

Abnormalities in apolipoproteins in PTC can be classified as particular types of altered whole‐body metabolic environments for cancer cells. Thyroid follicular epithelial cells are relatively rich in lipids in the cytoplasm due to their function of producing thyroid hormones; thus, they are more sensitive to changes in apolipoprotein levels (Lukasiewicz et al. 2024; Wan et al. 2025; Tu et al. 2025). Multi‐omics studies have shown that altered apolipoprotein levels are no longer passive consequences of PTC but actively promote dedifferentiation and metastasis (Xiao et al. 2023). Although static apolipoprotein alterations in established PTC have received some attention, the changes of these alterations over time in the DTI are unknown. In other words, it is unknown whether apolipoprotein dysregulation increases steadily with extended delay or whether there is a threshold‐dependent inflection point at a specific time boundary; therefore, a biologically informed ‘safe waiting period’ cannot be determined (Quail and Joyce 2013; Junttila and de Sauvage 2013). With the progress of next‐generation sequencing and the abundance of comprehensive transcriptomic data, large‐scale studies on the DTI‐metabolism axis have been conducted.

TCGA‐THCA and GEO offer multi‐omics data with fine resolution and a large number of samples, and can thus construct a model for treatment delay as a continuous molecular variable instead of using arbitrary binary stratification (Tu et al. 2025; Kim et al. 2021). TCGA‐THCA clinical data contain diagnosis and surgery timestamps; after harmonisation and quality control, these can be used to operationalise DTI as a quantitative exposure and a resource that has been relatively ignored in previous PTC biomarker studies (Li, Han, et al. 2025; Qiu et al. 2025). Most previous research on treatment delay has used administrative data from the SEER and NCDB databases, focused on endpoints such as pathological upstaging or survival, and has not explored the transcriptomic and lipidomic changes caused by DTI. Although the previous transcriptomic analysis has identified associations between gene expression and the disease, causality has not been confirmed due to the potential of confounding factors and reverse causation (Alnwisser et al. 2025). Mendelian randomization (MR) can be used to address the above issues by employing germline genetic variants as instrumental variables (IVs) and thus conducting less biassed causal inference from observational data (Sanderson et al. 2022). Integrate TCGA‐based discovery, GEO‐based external validation and genome‐wide association study (GWAS)‐driven MR to build a multi‐level evidence system that grounds molecular findings in population‐level transcriptomics, verifies their reproducibility across independent cohorts and determines etiological relevance through genetic randomization.

This study examines whether the delay of treatment in PTC causes different biological effects over time and how to identify a biologically rational safe observation period through dynamic changes in apolipoprotein dysregulation. An all‐encompassing analysis approach was employed in this study to cover TCGA‐THCA discovery, GEO validation, machine learning‐assisted gene prioritization and MR inference, in order to determine whether a biological safety window exists for PTC treatment delay and to develop a molecularly grounded risk stratification model for preoperative management that moves beyond arbitrary time cutoffs by identifying objective metabolic biomarkers.

## 2 Materials and Methods

### 2.1 Study Design and Analytical Framework

An iterative validation framework was used in this study to integrate transcriptomic discovery, cross‐cohort replication, interpretable machine learning and MR for molecular correlates of treatment delay in PTC. The four stages of the general design were: (1) discovery of treatment delay‐associated transcriptomic signatures in the TCGA cohort, and characterisation of linear relationships between core gene expression and delay duration using continuous variable modelling. This way, the progressive molecular changes and discrete threshold effects were differentiated. (2) Independently validated external verification of apolipoprotein dysregulation pathways in TCGA‐THCA across three GEO datasets. (3) Identification of key regulatory genes and quantification of their predictive contributions and synergistic interactions using SHapley Additive exPlanations (SHAP). (4) Genetic (Mendelian randomization) analysis of apolipoprotein‐trait associations with thyroid cancer risk. This modular design enables internal consistency checks at all levels of analysis: transcriptomic associations (Phases 1–2) are tested for predictive robustness (Phase 3), and subsequently, only after passing replication and interaction validation, are they examined for genetic support (Phase 4).

### 2.2 Data Acquisition and Preprocessing for the Discovery Cohort

Transcriptomic data (FPKM‐normalised RNA sequencing) and matched clinical annotations for PTC were obtained from TCGA‐THCA via the Genomic Data Commons (GDC) Data Portal. Of the 397 patients with clinical annotations, 285 had complete transcriptomic profiles and thus leaving 209 patients for integrated analysis of paired clinical‐molecular data. Exclusion criteria were incomplete clinical timelines, prior neoadjuvant therapy or non‐PTC histology. DTI is the time from the first abnormal examination to surgery for a person. DTI was dichotomised based on an empirically derived threshold for the first transcriptomic characterisation. A maximally selected rank statistic was used to find the optimal cut‐off that maximised the separation of postoperative complete response (CR) rates in the TCGA cohort. Patients were divided into a non‐delay and a delayed group solely for initial differential expression analysis; this threshold was exploratory and not intended to be clinically validated. GEO datasets were obtained for external validation (as shown below). The TCGAbiolinks package (v2.25.0) in R (v4.2.2) was used for quality control of the expression data. To reduce noise from low‐abundance transcripts, genes with a mean expression of less than 1 FPKM across the samples were excluded (Colaprico et al. 2016).

### 2.3 Differential Gene Expression and Functional Enrichment Analysis

Differentially expressed genes (DEGs) in the non‐delay and delayed groups were identified using limma with empirical Bayes moderation (eBayes) applied to log2‐transformed FPKM values in the R package limma (Ritchie et al. 2015). Genes were considered significantly differentially expressed if their absolute log_2_ fold change (FC) exceeded 1.0 and the Benjamini–Hochberg (BH) adjusted *p*‐value was less than 0.05. To identify the signalling pathways altered by the delay in treatment, Kyoto Encyclopaedia of Genes and Genomes (KEGG) pathway enrichment analysis was conducted on the DEGs using the clusterProfiler package (Wu et al. 2021). Pathways with an adjusted *p*‐value < 0.05 were regarded as significantly enriched. To find genes that belong to several delayed metabolic pathways, the gene sets of all significantly enriched KEGG pathways were taken and their intersection calculated with the UpSetR package (Conway et al. 2017). To determine whether apolipoprotein dysregulation exhibited a unified functional programme rather than a pathway‐specific abnormality, Gene Ontology (GO) enrichment was conducted on the entire set of differentially expressed genes (DEGs) (independently of KEGG intersections) across all three ontologies: Biological Process (BP), Cellular Component (CC) and Molecular Function (MF), using clusterProfiler and BH false discovery rate adjustment (adjusted *p* < 0.05). For clarity, the GO BP term ‘lipoprotein metabolic process’ (GO:0034381) will be referred to as ‘apolipoprotein‐associated metabolic process’ in the following text to highlight the target gene family.

### 2.4 Continuous Variable Modelling and SHAP‐Based Feature Attribution

To determine whether the connection between DTI and metabolic disorders was a linear relationship or had a threshold/inflection point, binary classification was replaced by continuous modelling. Apolipoprotein dysregulation‐associated genes identified by KEGG and GO analyses were chosen as the feature set. Normalised FPKM expression values were taken as the independent variables, and DTI (in days) was used as the dependent variable for linear regression. Model performance was shown by *R* ^2^ and root‐mean‐square error (RMSE). Genes with significant linear associations (BH‐adjusted *p* < 0.05) were considered DTI‐responsive genes.

To determine the effect of individual genes and investigate gene–gene interactions, a normalised FPKM expression matrix (X) and DTI values (Y) were used to build an XGBoost regression model with 10‐fold cross‐validation and grid search hyperparameter tuning. An interaction was considered structurally stable if it ranked among the top 10% of interaction magnitudes in at least 8 out of 10 folds (Chen and Guestrin 2016). SHAP was used to decompose feature importance, and the global SHAP values of each gene's contribution to DTI prediction were obtained (Lundberg and Lee 2017). SHAP interaction values (*φ*_*ij*_) were calculated to identify synergistic (non‐additive) effects of gene pairs, and interaction networks were constructed to visualize these pairwise relationships.

### 2.5 External Validation in Independent GEO Cohorts

For external validation, three independent GEO microarray datasets (GSE29265, GSE33630 and GSE60542) of case–control PTC transcriptomes were acquired. The selection of the above datasets meets the following criteria: (1) inclusion of primary PTC and matched adjacent normal thyroid tissue; (2) availability of processed microarray expression data; and (3) independent geographic and institutional origins to reduce cohort‐specific confounding.

Differential expression analysis of microarray intensity data were conducted using limma. DEGs were defined as genes with log_2_FC > 1 and BH‐adjusted *p* value < 0.05. GO enrichment and gene set enrichment analysis (GSEA) were conducted independently on each dataset using clusterProfiler (v4.5.1) with the KEGG and GO databases, and BP, CC and MF ontologies were considered. A dysregulation pathway of apolipoprotein reached marginal significance (*p* < 0.05) in at least two of the three cohorts and was thus regarded as consistent across cohorts. UpSet analysis identified gene overlaps of the DEGs, and a random‐effects model was employed to address heterogeneity.

The TCGA pipeline annotates the apolipoprotein C gene family as a whole under APOC, but GEO datasets resolve individual family members (APOC1, APOC2, APOC3). As shown in the sequence ID cross‐mapping, the APOC designation of the TCGA temporal prediction framework corresponds to APOC1 in the GEO disease‐state framework.

### 2.6 Machine Learning‐Based Evaluation of Core Gene Predictive Performance and Gene–Gene Interactions

Intersection analysis of DEGs consistently enriched in all three GEO datasets to produce a candidate consensus gene set. To assess the combined predictive power of these hub genes for disease status, normalised expression values of the hub genes were used to construct a feature matrix (X), and disease status (tumour vs. normal) served as the binary response variable (Y). A 10‐fold cross‐validation was used to train an XGBoost classifier for the predictive architecture. Rank the importance of each gene in classifying the disease using SHAP values. Gene–gene interaction analysis was then carried out to find joint (non‐additive) effects; pairwise interaction terms were added to the regression model, and the relative contribution of each interaction to the predictive variance was quantified.

### 2.7 Two‐Sample Mendelian Randomization

A two‐sample Mendelian Randomization analysis was conducted to determine if genetically predicted circulating lipoprotein and metabolite traits were associated with the risk of PTC, and the results followed the STROBE‐MR guidelines (Skrivankova et al. 2021). MR is based on three assumptions: the instrument is related to the exposure (relevance), it does not have a common cause with the outcome (independence), and it only affects the outcome through the exposure (exclusion restriction). Due to the transcriptomic analysis indicating that triglycerides were involved in VLDL‐related lipoprotein metabolism, we defined VLDL subfraction traits and HDL cholesterol as contrasting traits, together with a pre‐specified set of primary interests based on biological reasons, and then examined the results of the genetic association study for these traits; other traits were treated as secondary, exploratory screens.

#### 2.7.1 Exposure Data

Genetic associations of 233 circulating metabolic biomarkers, including lipoprotein particle concentrations and subclasses measured by nuclear magnetic resonance (NMR) spectroscopy, were obtained by Karjalainen et al. (2024). A total of 136,016 participants of European ancestry across 33 cohorts are included in this dataset.

#### 2.7.2 Outcome Data

Genetic associations with thyroid cancer were derived from the two independent GWAS of European‐ancestry individuals. The first outcome dataset is EBI OpenGWAS study GCST90018929 (ebi‐a‐GCST90018929; 1054 cases and 490,920 controls; total *n* = 491,974). A smaller independent dataset, IEU‐A‐1082 (649 cases and 431 controls; total *n* = 1080), was used to examine directional consistency, but due to severe underpowering, no primary results could be obtained from this set. mRnd (https://shiny.cnsgenomics.com/mRnd/) was employed to determine the statistical power. Power exceeded 99% in the main outcome for detecting a causal effect of *β* = 0.2 at *α* = 0.05. The small replication set (*n* = 1080) lacked statistical power for a weak effect, as mentioned in the interpretation.

#### 2.7.3 IV Selection

Instruments were selected at a significance threshold of *p* < 1 × 10^−5^, and they had to be available for the traits with few genome‐wide significant variants (Yao et al. 2026). Linkage disequilibrium clumping was then performed with the 1000 Genomes European reference panel (*r* ^2^ < 0.001 in a 10,000 kb window), and the variant with the smallest *p* value in each clump was retained. Exposure and outcome datasets were harmonised to a common effect allele, and palindromic SNPs with intermediate allele frequencies were excluded to avoid ambiguous strand alignment. Given that instruments were selected at *p* < 1 × 10^−5^, each instrument has, by construction, a minimum *F*‐statistic (calculated as *β* ^2^/SE^2^) of about 19.5, and all instruments thus exceeded the traditional *F* > 10 threshold for guarding against weak‐instrument bias (Burgess and Thompson 2021).

#### 2.7.4 Causal Effect Estimation

The first is an inverse‐variance weighted (IVW) method under a multiplicative random‐effects model; for exposures instrumented by a single SNP, a Wald ratio was used. Estimates are reported as the change in the log‐odds of PTC (*β*) per standard deviation increase in the genetically predicted trait, along with 95% confidence intervals. As complementary estimators that make different assumptions about the validity of the instrument, MR‐Egger regression, the weighted median, the weighted mode and the simple mode were also calculated for each exposure and reported along with the IVW estimate (Bowden et al. 2017).

#### 2.7.5 Correction for Multiple Testing

In the predefined VLDL set, the threshold for statistical significance was Bonferroni corrected (0.05 divided by the number of traits in the set). Across all 233 traits in the full‐screen display, the Benjamini–Hochberg procedure was used to control the false discovery rate at 5%, and both nominal *p* values and FDR‐adjusted *q* values were provided for every exposure. The two were analysed separately on the primary (GCST90018929) and replication (ieu‐a‐1082) sets.

Sensitivity analysis: For each exposure, one‐way directional horizontal pleiotropy was tested using the MR‐Egger intercept test (*p* < 0.05), and thus pleiotropy was identified. Cochran's *Q* statistic is used to determine whether there is heterogeneity in the results of the IVW and MR‐Egger models (Verbanck et al. 2018). All the analyses were conducted in R with the TwoSampleMR package, and LD clumping used the 1000 Genomes European reference panel.

## 3 Results

### 3.1 Identification of a 77‐Day Inflection and Apolipoprotein Dysregulation in TCGA‐THCA

**FIGURE 1:**
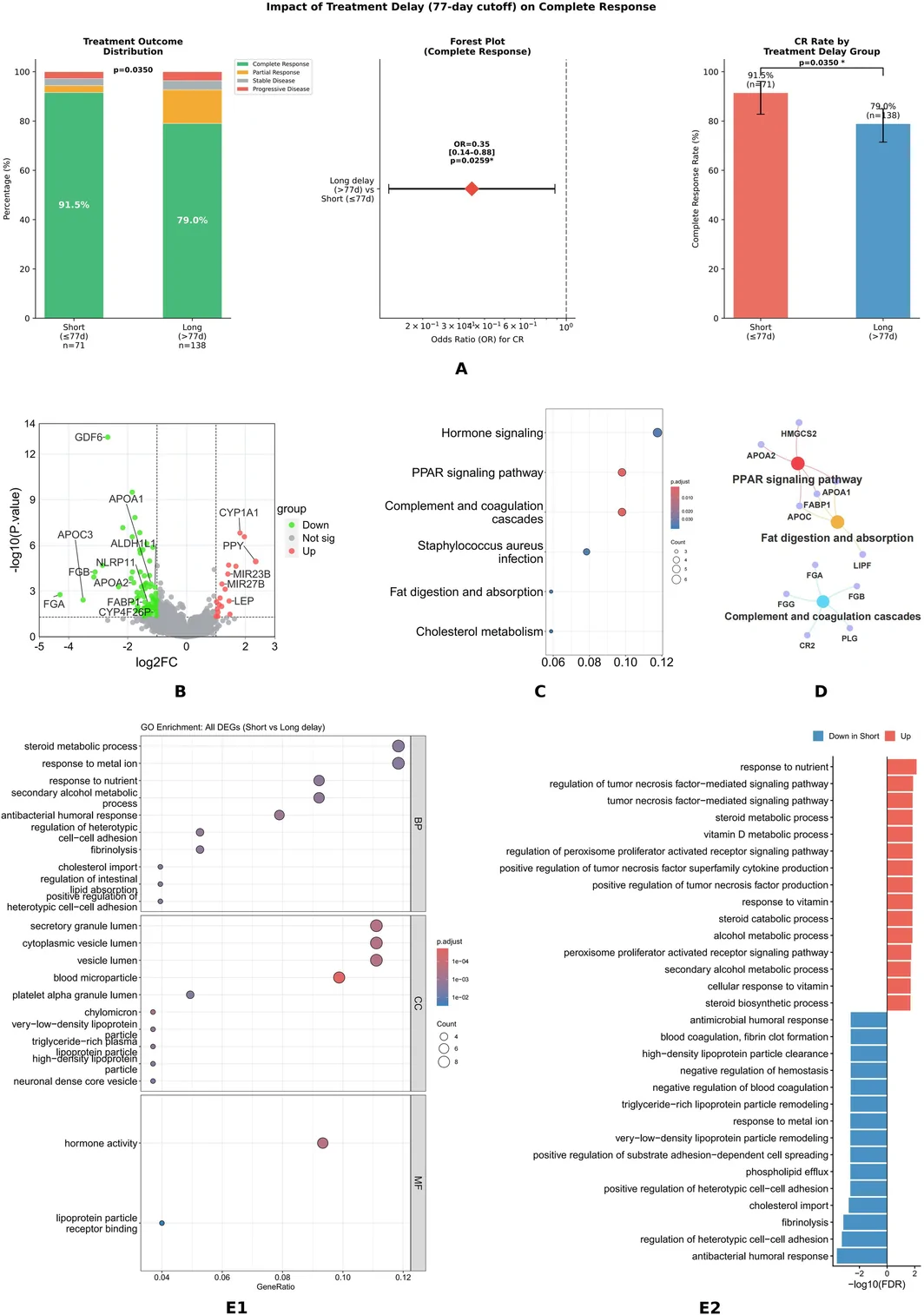
Treatment delay‐associated clinical and transcriptomic alterations in TCGA‐THCA. (A) CR rates stratified by DTI group (≤ 77 vs. > 77 days; *n* = 209). (B) Volcano plot showing DEGs: Blue indicates upregulated genes in long‐delay; red indicates downregulated genes (|log_2_FC| > 1, FDR < 0.05). APOC in TCGA corresponds to APOC1 (HGNC:601). (C) KEGG pathway enrichment of DEGs, with three pathways reaching FDR < 0.05. (D) Pathway intersection analysis (PPAR ∩ Complement ∩ Fat digestion) identifies APOA1, APOC and FABP1 as the shared apolipoprotein triad connecting PPAR signalling and fat digestion and absorption. (E1–E2) GO enrichment visualized across BP, CC and MF ontologies. Dot size represents gene count; colour indicates adjusted *p* value.

In the TCGA‐THCA discovery cohort (*n* = 209), a 77‐day threshold was set, and the CR rates of the treatment‐delayed group and others differed significantly. Patients who received definitive surgery within 77 days of pathological diagnosis had a CR rate of 91.5% and a lower rate of 79% for those with DTI exceeding 77 days (*p* = 0.0350) (Figure 1A). At this threshold, the complete‐response rate exceeded 50% in the cohort.

Differential expression analysis showed that 77 DEGs (|log_2_FC| > 1.0, BH adjusted *p* < 0.05) were present, of which 61 were upregulated (blue set in the volcano plot) and 16 were downregulated (red set) in the delayed group (> 77 days vs. ≤ 77 days) (Figure 1B). The directionality is based on the limma (eBayes) contrast, and the short‐delay group is taken as the baseline.

Among these DEGs, 51 mapped to KEGG pathways (mapping rate: 66.2%). Enrichment analysis identified three pathways that were significantly enriched in the delayed group: PPAR signalling (5/51, *p* = 1.13 × 10^−4^, adjusted *p* = 9.48 × 10^−3^; APOA2, APOA1, APOC, FABP1, HMGCS2), complement and coagulation cascades (5/51, *p* = 1.77 × 10^−4^, adjusted *p* = 9.48 × 10^−3^; FGB, FGG, CR2, PLG, FGA), and fat digestion and absorption (3/51, *p* = 1.69 × 10^−3^, adjusted *p* = 3.62 × 10^−2^; APOA1, FABP1, LIPF, APOC) (Figure 1C). PPAR signalling and fat digestion pathways are both associated with apolipoprotein family members, and they can occur simultaneously. Pathway intersection analysis identified APOA1, APOC and FABP1 as the common apolipoprotein triad of these pathways (Figure 1D). These genes code for the structural scaffold (APOA1), lipolytic regulator (APOC), and intracellular lipid chaperone (FABP1) of triglyceride‐rich apolipoproteins, and their coordinated dysregulation affects the dynamics of lipoprotein particles in the delayed‐treatment setting.

GO enrichment analysis of the upregulated DEG subset (*n* = 61) supported an apolipoprotein‐centric activation signature. The delayed group showed significant enrichment in processes associated with apolipoprotein particle dynamics, including high‐density lipoprotein particle clearance (*p* = 6.76 × 10^−6^, adjusted *p* = 2.52 × 10^−3^; APOA2, APOA1, APOC3), triglyceride‐rich lipoprotein particle remodelling (*p* = 9.27 × 10^−6^, adjusted *p* = 2.52 × 10^−3^; APOA2, APOA1, APOC3), very‐low‐density lipoprotein particle remodelling (*p* = 9.27 × 10^−6^, adjusted *p* = 2.52 × 10^−3^), phospholipid efflux (*p* = 1.60 × 10^−5^, adjusted *p* = 2.92 × 10^−3^; APOA2, APOA1, APOC3), reverse cholesterol transport (*p* = 3.10 × 10^−5^, adjusted *p* = 3.39 × 10^−3^; APOA2, APOA1, APOC3), high‐density lipoprotein particle remodelling (*p* = 3.76 × 10^−5^, adjusted *p* = 3.39 × 10^−3^; APOA2, APOA1, APOC3), and negative regulation of lipase activity (*p* = 1.54 × 10^−3^, adjusted *p* = 2.87 × 10^−2^; APOA2, APOC3) (Figure 1E). Convergence of biological process (lipid transport), cellular component (apolipoprotein particles), and molecular function (lipase inhibition and cholesterol efflux) ontologies suggests that these transcriptional changes are a targeted apolipoprotein‐mediated dysregulation programme rather than a non‐specific stress response.

### 3.2 Continuous Model: Apolipoprotein Expression Is Linearly Related to the Duration of DTI

Linear regression models of DTI showed that apolipoprotein expression increases gradually with longer delays. APOA1 and APOC have significantly positive coefficients; thus, each extra day of delay progressively increases apolipoprotein dysregulation, and therefore prolonged DTI leads to worsening molecular alterations along a linear path.

**FIGURE 2:**
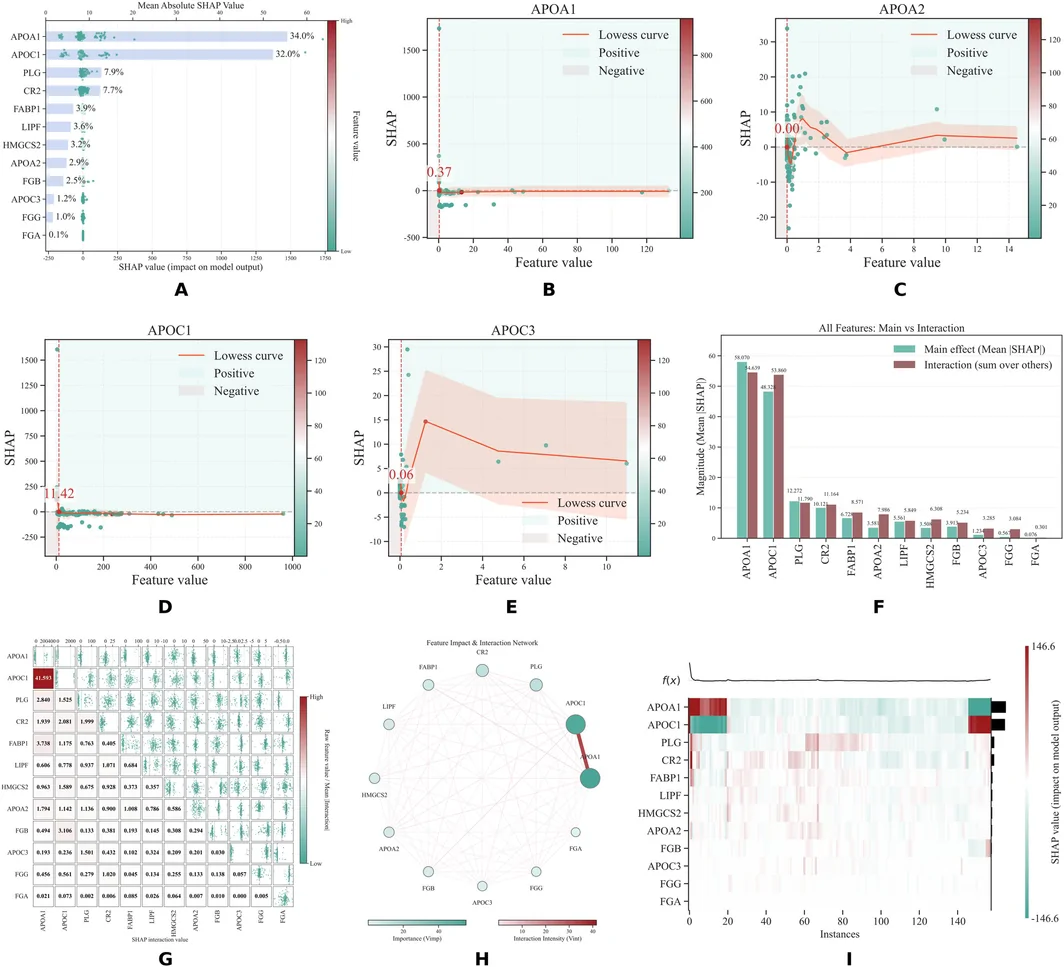
Continuous modelling and interpretable machine learning reveal a linear time–expression relationship and APOA1–APOC joint‐contribution architecture in treatment delay. (A) Global SHAP feature importance ranking for the XGBoost regression model. (B–E) SHAP dependence plots for APOA1, APOA2, APOC1 and APOC3, respectively, showing normalised FPKM expression on the *x*‐axis and SHAP values (in days) on the *y*‐axis, with near‐linear monotonic trends. (F) SHAP interaction contribution plot for apolipoprotein gene pairs. (G) SHAP interaction heatmap showing pairwise interaction strengths. (H–I) SHAP interaction network, where node size indicates global importance and edge thickness corresponds to mean absolute SHAP interaction. APOA1‐APOC edge is the main high‐confidence link.

An XGBoost regression ensemble was used to learn the non‐linear predictive relationship. Global SHAP analysis showed that APOA1 and APOC were the main contributors to variance globally, accounting for 66% of the total, far exceeding other features, and thus were identified as the leading molecular drivers of DTI prediction (Figure 2A).

SHAP dependence plots of the top four predictors—APOA1, APOA2, APOC1 and APOC3—showed near‐linear monotonic increases in predicted delay with rising expression levels, supported the regression results, and revealed slight saturation effects at the extremes of expression (Figure 2B–E). APOA1 and APOC3 had the steepest slopes and were therefore more significantly contributed by SHAP.

Interaction analysis revealed a gene–gene cooperative architecture, and the APOA1–APOC axis emerged as the main joint‐contribution module (Figure 2F). The interaction heatmap showed a strong co‐expression (collinearity) of APOA1 and APOC, and the mean of |SHAP interaction values| was significantly larger than that of any other gene pair (Figure 2G).

The interaction network showed that this topology was prominent: the APOA1–APOC edge stood out from the secondary connections and accounted for the main joint (non‐additive) contribution (Figure 2H,I). At high co‐expression levels, their combined predictive effect exceeded the sum of the individual marginal contributions; thus, they were not independently additive but rather jointly non‐additive. Based on the above results, the apolipoprotein abnormalities related to treatment delay are the result of a network effect and not caused by a single gene. Therefore, the APOA1–APOC axis acts as a regulatory centre to coordinate the dysregulation of apolipoproteins and increase metabolic vulnerability over time. As APOA1 and APOC1 are highly co‐expressed, this SHAP interaction should be regarded as their combined effect on the model rather than as mechanistic epistasis.

### 3.3 Cross‐Cohort Validation of Apolipoprotein Dysregulation in Independent GEO Datasets

**FIGURE 3:**
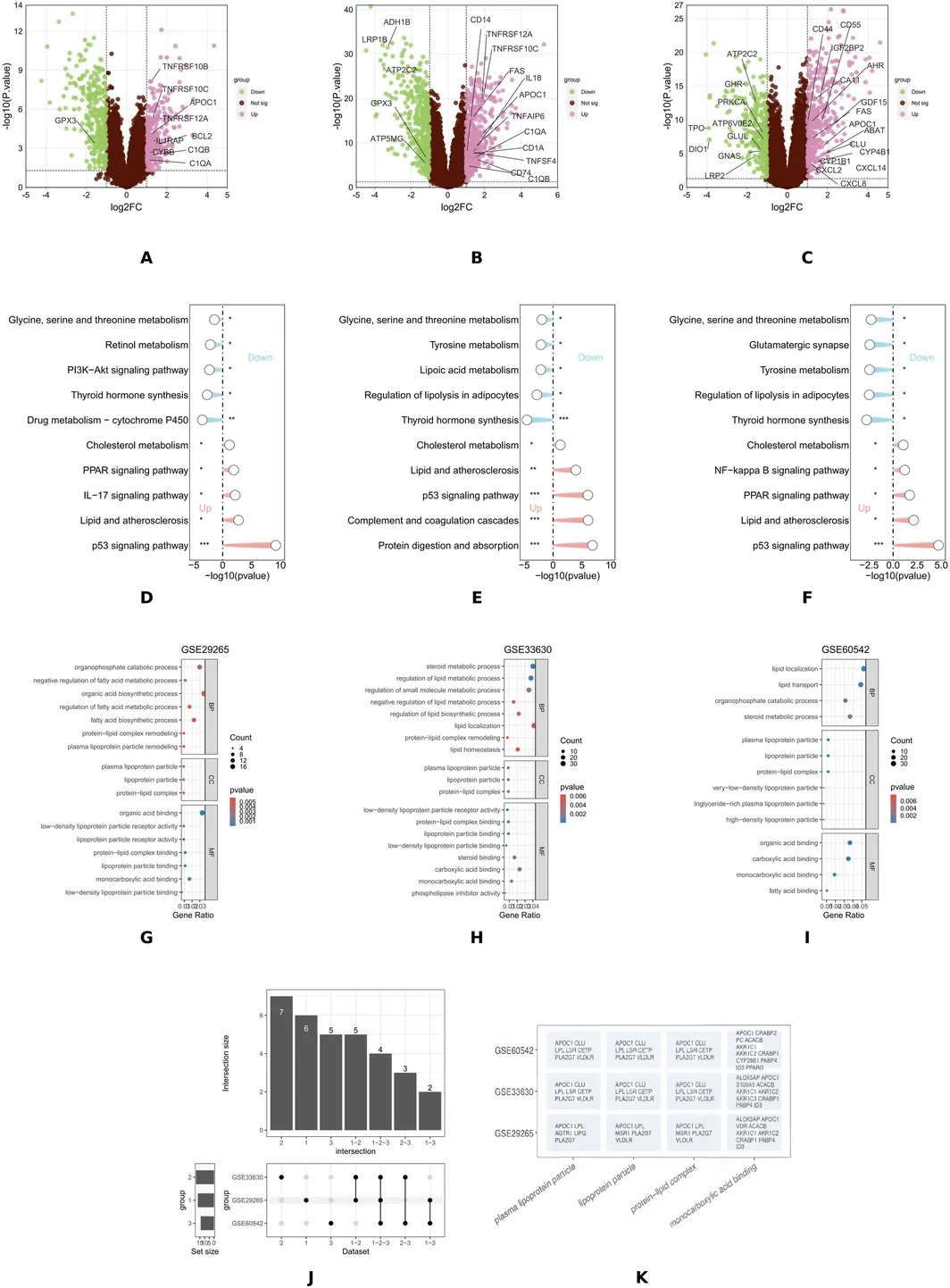
Cross‐cohort validation in independent GEO datasets confirms conserved apolipoprotein dysregulation in PTC. Volcano plots of differentially expressed genes (DEGs) comparing PTC tumors to adjacent normal tissue in three validation cohorts. Gene Ontology (GO) enrichment analyses of DEGs in GSE29265, GSE33630 and GSE60542. UpSet plots identifying consensus DEGs across discovery and validation datasets, highlighting a core set of five genes (LPL, APOC1, ID3, CLU and VLDLR) consistently dysregulated across GEO cohorts.

The reproducibility of the TCGA‐THCA‐derived transcriptomic signature was tested in three independent GEO PTC cohorts: GSE29265, GSE33630 and GSE60542. Differential expression analysis using the same limma‐based thresholds (|log_2_FC| > 1.0, adjusted *p* < 0.05) identified APOC1, LPL and VLDLR as the most consistently altered lipid‐metabolic genes. APOC1 was uniformly upregulated in all cohorts (GSE29265: log_2_FC = +1.16, adjusted *p* = 0.002; GSE33630: log_2_FC = +1.85, adjusted *p* < 0.001; GSE60542: log_2_FC = +1.71, adjusted *p* < 0.001), and LPL was also elevated (log_2_FC range +1.05 to +2.53, all adjusted *p* < 0.05) (Figure 3A–C). On the other hand, VLDLR was significantly downregulated in all the datasets (log_2_FC range −1.09 to −1.41, all adjusted *p* < 0.01), suggesting that lipoprotein particle clearance was suppressed throughout the delayed‐treatment tumour microenvironment.

KEGG pathway enrichment further supported the previous results and showed consistent upregulation of cholesterol metabolism, lipid and atherosclerosis, and PPAR signalling pathways in GSE29265 and GSE60542, as well as p53 signalling as a common oncogenic stress response across all three cohorts (Figure 3D–F). Downregulated pathways uniformly included thyroid hormone synthesis and regulation of lipolysis in adipocytes, indicating a widespread suppression of thyroid‐specific metabolic regulation and peripheral lipolytic abnormalities.

GO enrichment further supported the apolipoprotein‐centred functional architecture of all three GEO datasets. The upregulated DEGs were significantly enriched in CCs related to plasma lipoprotein particles and lipoprotein particles, BPs involved in lipid localisation and lipid transport, and MFs such as lipoprotein particle binding (Figure 3G–I). Notably, the dataset GSE60542 uniquely showed enrichment in very‐low‐density lipoprotein particle and triglyceride‐rich plasma lipoprotein particle components, directly indicating that the VLDL metabolic axis was involved in the cross‐cohort transcriptomic signature.

The GO intersection matrix identified key gene drivers for the above functional terms (Figure 3K). APOC1 was the only gene present in all 12 intersection cells (4 GO terms × 3 datasets) and thus considered a high‐confidence cross‐cohort hub. LPL co‐occurred with APOC1 in the plasma lipoprotein particle, lipoprotein particle, and protein–lipid complex terms of all three datasets, but VLDLR was not present in GSE29265 for the first two terms and reappeared in the protein–lipid complex category. The monocarboxylic acid binding term added the genes for FABP4 and PLA2G7, suggesting that the activity of intracellular lipid chaperones is also required for the clearance of extracellular lipoproteins.

Among them, ID3 and CLU showed partial but consistent cross‐cohort patterns. ID3 was significantly downregulated in all datasets (log_2_FC = −1.15 to −1.37, adjusted *p* < 0.001) and is known to suppress lipolysis. CLU was significantly upregulated in GSE33630 and GSE60542 (log_2_FC = +1.51 and +1.87), and appeared in lipoprotein‐related GO terms in these cohorts; however, it showed a non‐significant fold change in GSE29265 (log_2_FC = +0.42, adjusted *p* = 0.55), which is likely due to differences in microarray platforms (Affymetrix U133A vs. U133 Plus 2.0). Upset intersection analysis of the significantly enriched GO pathways quantified the extent of cross‐cohort convergence: four GO terms were shared by all three datasets, as well as pairwise overlaps of five terms (GSE29265–GSE33630), three (GSE33630–GSE60542) and two (GSE29265–GSE60542) terms (Figure 3J).

Based on the above results, the apolipoprotein dysregulation in the TCGA discovery cohort has been validated as a reproducible transcriptional program across multiple independent PTC cohorts. The APOC1‐LPL‐VLDLR axis is the core conserved hub, and ID3 always marks a suppressed lipolytic regulator.

### 3.4 Disease‐State Predictive Modelling Confirms the Five Consensus Genes as Functional Drivers of PTC

**FIGURE 4:**
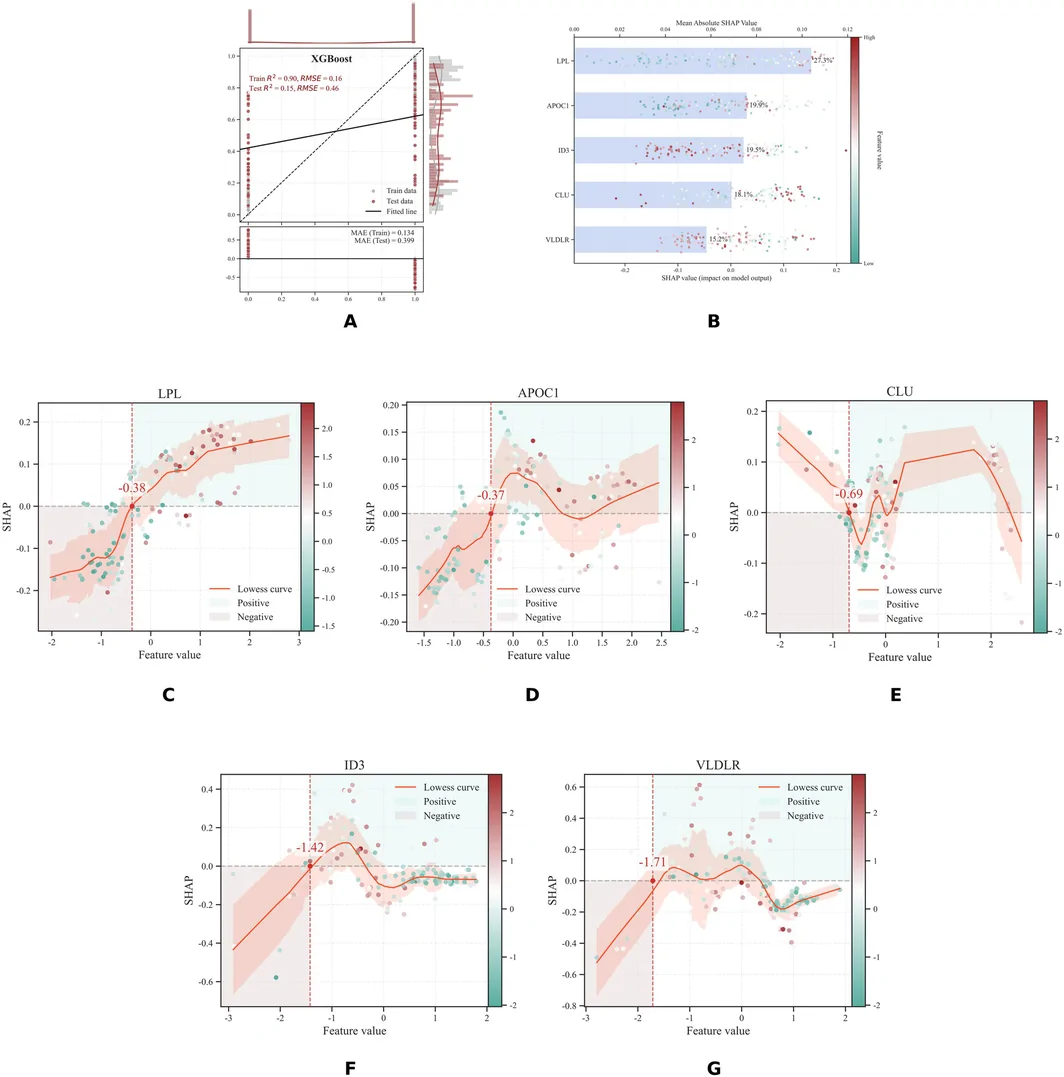
The XGBoost‐SHAP predictive architecture of the five‐gene consensus module for disease‐state classification. (A) The visualization of XGBoost model performance highlights prediction residuals, with cross‐validated *R* ^2^ and RMSE values indicated. (B) Global SHAP feature importance is ranked, demonstrating that all five consensus genes (LPL, APOC1, ID3, CLU, VLDLR) possess substantial predictive weight, with LPL and APOC1 exhibiting the highest individual fidelity. (C–G) SHAP dependence plots for (C) LPL, (D) APOC1, (E) CLU, (F) ID3 and (G) VLDLR illustrate normalised expression on the x‐axis and SHAP value in disease‐state probability on the *y*‐axis. A near‐linear monotonic increase is evident across the expression range, with LPL and APOC1 showing the steepest slopes.

An XGBoost classification model was employed to predict the status of the tissue (tumour or adjacent normal) based on the normalised FPKM expression data, and five cross‐cohort candidate genes (LPL, APOC1, ID3, CLU, VLDLR) were selected as features after differential expression and GO convergence analysis across the three GEO datasets. Cross‐validation (Figure 4A) showed that the five‐gene expression matrix has good discriminatory power for PTC malignancy and is not random in prediction. The above structure shows whether the gene set as a whole codes for a disease‐state signal in XGBoost, and SHAP explains the contribution hierarchy of individual genes.

Global SHAP feature importance decomposition shows that all five genes contribute to the classification in a meaningful way and none are below the noise threshold. LPL and APOC1 were the strongest individual predictors and main effectors, and ID3, CLU and VLDLR showed non‐redundant but weaker influences (Figure 4B). Therefore, the consensus signature is a coordinated multi‐gene predictive network rather than the result of a single‐gene effect.

SHAP dependence plots for each gene (Figure 4C–G) showed expression‐dependent modulation of the predicted disease probability. LPL and APOC1 showed the steepest monotonic increases in the range of observed expression, consistent with their dominant SHAP contributions, and thus were considered an integrated predictive unit rather than independent biomarkers.

**FIGURE 5:**
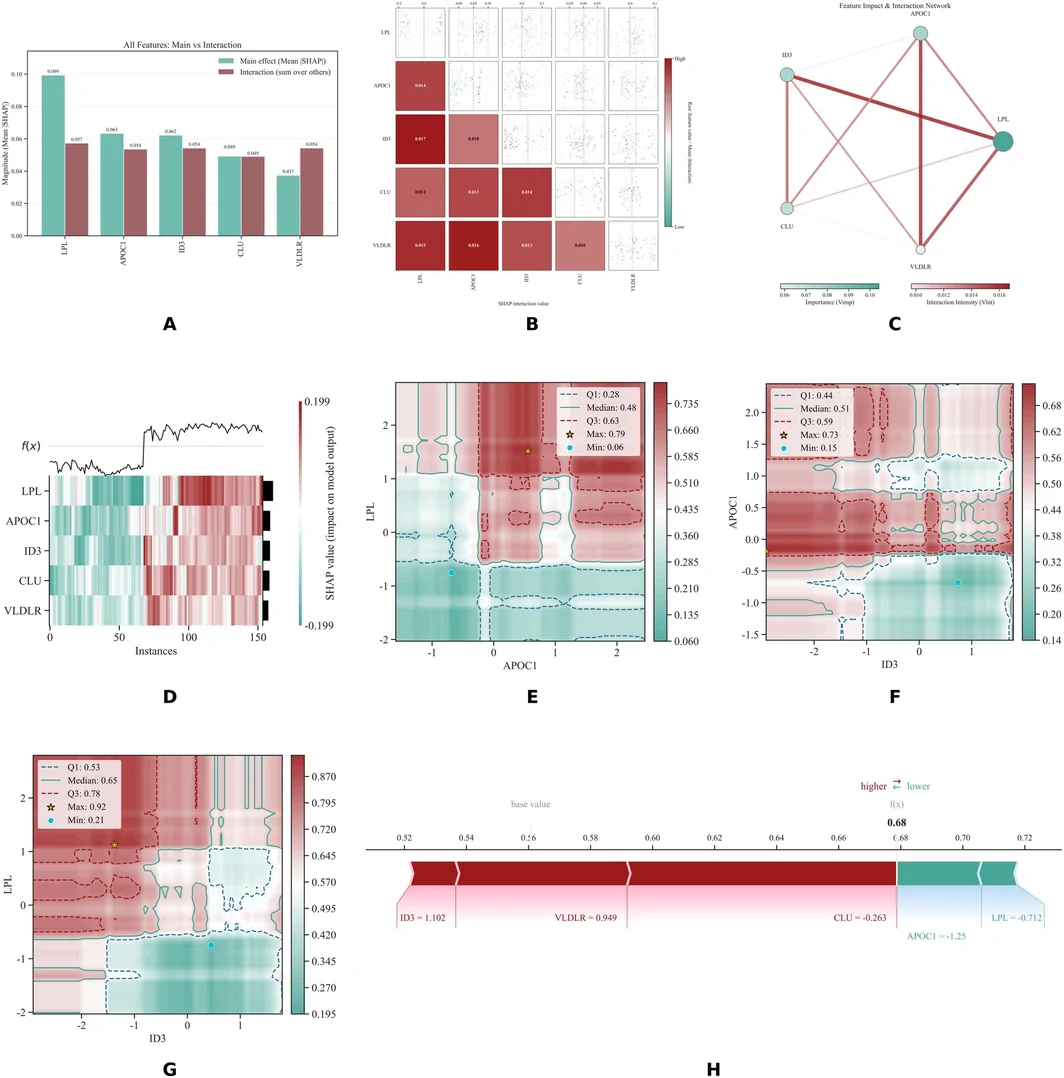
SHAP interaction analysis, revealing LPL–APOC1 as the joint‐contribution axis within the five‐gene module. (A) The SHAP interaction contribution plot depicts the main effect versus interaction effect for all pairwise combinations, with the LPL–APOC1 interaction dominating the interaction landscape. (B) The combined interaction matrix integrates main effects and interaction weights across the five‐gene module. (C) A SHAP interaction network is shown, where node size reflects global feature importance, and edge thickness represents the mean absolute SHAP interaction value. The LPL–APOC1 edge serves as the primary high‐confidence connection. (D) A SHAP interaction heatmap quantifies pairwise joint‐contribution strengths. (E) An interaction scatterplot (APOC1 vs. LPL) reveals that high co‐expression levels yield joint predictive signals that surpass additive expectations. (F, G) Secondary interaction scatterplots for (F) ID3 vs. APOC1 and (G) ID3 vs. LPL remain below the synergistic threshold. (H) A representative SHAP force‐plot decomposition for an individual tumour sample illustrates the collective influence of the five genes on disease‐state prediction.

SHAP interaction analysis identified gene–gene cooperative topology. The interaction contribution plot showed that the LPL‐APOC1 pair was the primary joint‐contribution axis and significantly higher than all other combinations (Figure 5A). The interaction matrix, network graph and heatmap consistently showed that LPL‐APOC1 was the dominant joint‐contribution connection; interactions involving ID3, CLU and VLDLR were close to the background (Figure 5B–D). Interaction scatterplots show that at higher co‐expression levels, the combined predictive effect of LPL and APOC1 exceeds the sum of their individual marginal effects; thus, it is a joint, non‐additive contribution rather than simple addition (Figure 5E). Secondary interactions, such as ID3‐APOC1 and ID3‐LPL, were below the interaction threshold (Figure 5F,G). A representative SHAP force plot further shows that the combined action of these five genes is responsible for the disease‐state prediction at the level of individual samples (Figure 5H).

APOC1 is the only gene that connects the TCGA temporal‐prediction framework with the GEO disease‐state framework. APOA1 and LPL are involved in other circumstances at different times or in different disease states. The above complementary and non‐redundant architectures indicate that apolipoprotein dysregulation occurs along convergent paths in PTC.

### 3.5 Two‐Sample Mendelian Randomization of Circulating Metabolic Traits and PTC Risk

To determine if the lipid‐metabolic alterations identified by transcriptomic analysis are genetically predicted drivers of PTC, two‐sample MR was conducted for 233 NMR‐quantified metabolic traits against the two independent thyroid cancer GWAS. The first analysis used the large outcome dataset (GCST90018929; *n* = 491,974), and the smaller dataset (ieu‐a‐1082; 649 cases) was employed for direction consistency tests.

In the primary dataset, all 233 exposures were included in the nominal significance set of the inverse‐variance weighted (IVW) estimate (*p* < 0.05), and 36 of these were in the replication dataset. After correction for multiple testing, none of these associations remained significant under Benjamini–Hochberg control (smallest *q* = 0.37 in the primary and 0.30 in the replication dataset) or under Bonferroni correction, and none survived Bonferroni correction within the pre‐specified VLDL set (*k* = 75). The nominally associated traits in the two datasets were largely non‐overlapping. The largest individual estimate, triglycerides in very‐large VLDL in the replication dataset (IVW *β* = 1.48; 95% CI 0.30 to 2.67; *p* = 0.014), was not reproduced in the larger dataset (*β* = 0.15; *p* = 0.19) and did not survive correction in either.

**FIGURE 6:**
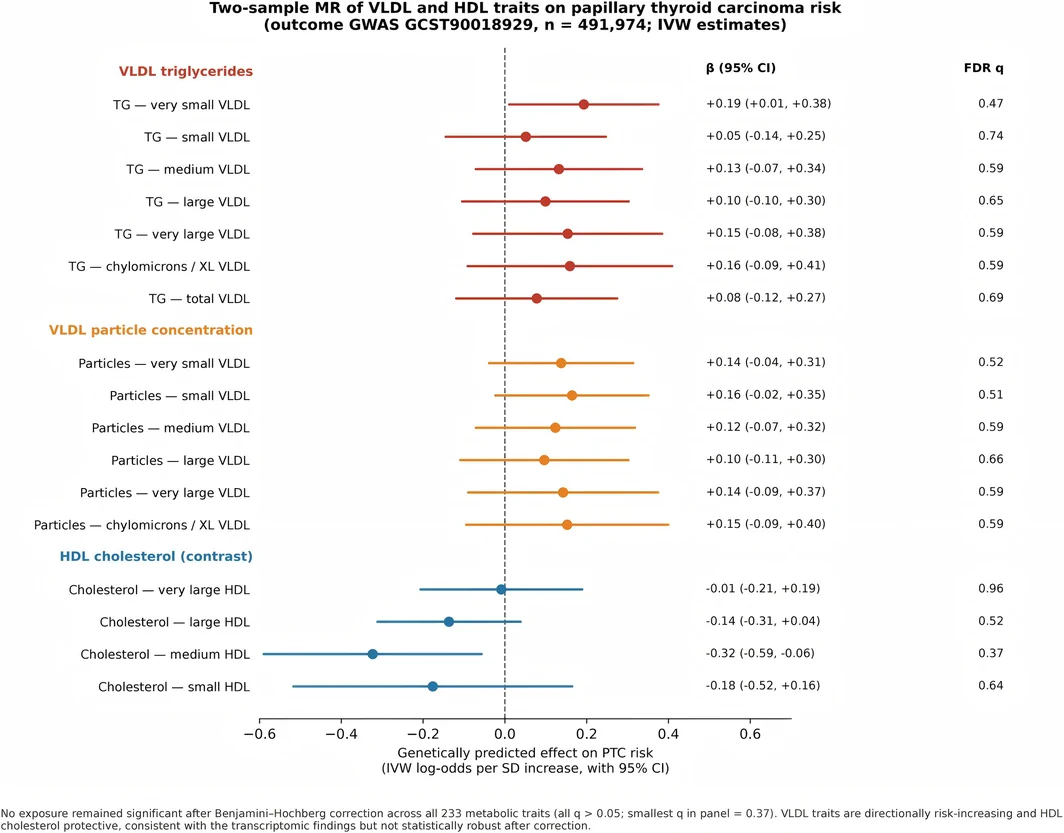
Two‐sample Mendelian randomization of genetically predicted VLDL and HDL traits on papillary thyroid carcinoma risk, using the larger thyroid cancer GWAS (GCST90018929; *n* = 491,974). Points are inverse‐variance weighted estimates (log‐odds of PTC per SD increase in the genetically predicted trait); horizontal lines are 95% confidence intervals; the dashed line marks the null. FDR *q* values are Benjamini–Hochberg‐adjusted across all 233 metabolic traits. VLDL triglyceride and particle measures were directionally associated with increased risk and HDL cholesterol with decreased risk, mirroring the transcriptomic findings, but no exposure remained significant after correction for multiple testing (all *q* > 0.05). Full results for all 233 traits are in Table S1.

None of the individual traits were statistically significant, but the direction of the effect for related traits was consistent. In the primary dataset, 59 out of 75 VLDL subfraction traits showed a positive, risk‐increasing point estimate (Figure 6), and 21 out of 24 HDL cholesterol traits were negatively correlated with risk and protective, respectively; this is consistent with the up‐regulation of VLDL‐handling genes and the HDL‐clearance signature observed in the transcriptomic analysis. Because of the strong correlation among these subfraction traits, they likely originate from a few fundamental signals rather than independent replications, and thus should not be overemphasised.

Evidence of directional pleiotropy was rare (MR‐Egger intercept *p* < 0.05 for 2 of the 232 traits in the primary dataset and 5 of the 231 traits in the replication dataset), and between‐instrument heterogeneity was moderate (Cochran's *Q p* < 0.05 for 17 and 18 traits, respectively). The estimates of the apolipoprotein B to apolipoprotein A1 ratio were inconsistent in the two datasets (*β* = 0.19 vs. *β* = −1.29), and they could not be reconciled; therefore, this composite index was not further analysed.

In combination, the MR analysis is in the same direction as the transcriptomic data, but after correction it has not reached statistical significance. Therefore, we consider this to be supportive and hypothesis‐generating work, and the main conclusions of this study are based on the reproducible cross‐cohort transcriptomic signature.

## 4 Discussion

We delineate the inferential boundaries of the multi‐phase design. Transcriptomic analysis shows an association between extended DTI and altered apolipoprotein distribution in PTC. At the same time, MR analysis was conducted to determine if genetically predicted apolipoprotein‐related metabolic traits were associated with thyroid cancer susceptibility; after correcting for multiple testing, it did not provide strong causal support, although the direction of the effect was consistent with that found in transcriptomics. Although both lines of evidence converged on the apolipoprotein‐VLDL axis, they did not present a single causal pathway: MR did not explore whether DTI was linked to apolipoprotein dysregulation, and the transcriptomic data did not show that the observed expression changes directly promoted malignancy. Observational co‐variation (DTI‐expression), cross‐cohort predictive robustness (GEO‐SHAP), and genetic causal inference (MR) were used instead to provide convergent evidence that strengthened the plausibility of the biological mechanism, but it was also determined that the DTI → dysregulation → PTC progression triad still requires prospective clinical validation.

### 4.1 Mechanistic Interpretation: The APOC1‐LPL Axis as a Potential Rate‐Limiting Step

XGBoost‐SHAP identified APOC1 as the conserved hub for connecting temporal delay and disease‐state discrimination, which exhibited strong interaction effects with LPL. The close statistical association of APOC1 and LPL is consistent with their known biochemical linkage; given that they are co‐expressed, it should be interpreted as a combined predictive effect rather than evidence of epistasis. APOC1 codes for apolipoprotein C‐I, an endogenous inhibitor of lipoprotein lipase that competes with the APOC2 cofactor for binding to the lipid‐water interface of triglyceride‐rich lipoproteins (Rouland et al. 2022). When APOC1 was overexpressed compared with APOC2, LPL‐mediated hydrolysis of VLDL core triglycerides was inhibited, and an enzymatic bottleneck arose in particle clearance.

Given the prolonged treatment delay, transcriptomic data have indicated that PTC cells can use this bottleneck for a double adaptation: upregulating the enhanced receptor‐mediated uptake of particles via VLDLR and reducing local lipolysis by raising APOC1 levels to retain lipid cargo in the tumour microenvironment. Therefore, there was a passive accumulation of VLDL in the blood, as well as an active diversion of lipoprotein flux to the tumour. This indicates that, in recent years, researchers have begun to study how solid tumours alter lipoprotein metabolism through increased de novo lipogenesis and enhanced scavenging of circulating lipid stores (Röhrig and Schulze 2016; Kuemmerle et al. 2011).

The MR results were in the same direction as this model but did not confirm it: genetically predicted VLDL and triglyceride‐rich lipoprotein traits were associated with an increased risk of PTC, although no individual association survived correction for multiple testing. On the other hand, the protective effects of HDL and IDL cholesterol subfractions suggest that the damage caused by cancer is compartment‐specific; namely, it is the triglyceride‐rich, APOC1‐regulated VLDL pool, rather than cholesterol transport itself, that is most consistently affected.

### 4.2 Treatment Delay and Oncologic Outcomes: Beyond Indolence

PTC has been known as an indolent disease for a long time, and a slight delay in treatment was considered harmless. However, new population‐level data have begun to overturn this idea. A meta‐analysis of 1.27 million patients with seven types of solid tumours showed that for every 4‐week delay in curative surgery, there was an increase of 6%–8% in mortality, and no safe threshold below 4 weeks could be determined (Hanna et al. 2020). Based on the SEER analysis of PTC, longer surgical delays disproportionately affected older, unmarried and rural patients, and were associated with an increased risk of non‐cancer death even if the cancer‐specific survival rates were the same (Zhang et al. 2024).

Our research added a biological basis to the above epidemiological data. If the accumulation of VLDL due to treatment delay is indeed causal, then the ‘safe waiting’ model for PTC may need to be stratified by metabolic phenotype. Patients with pre‐existing dyslipidaemia or a family history of VLDL accumulation may be at a higher risk for harm due to a prolonged delay in treatment, even if the tumour histology is low‐risk (Ping et al. 2025; He et al. 2022).

### 4.3 Mendelian Randomization Evidence and Its Limitations

After correcting for multiple tests, MR analysis did not provide strong support for a causal role of VLDL subfractions in PTC pathogenesis. The direction of the effect is in line with what was found in the transcriptomics, but it should be considered hypothesis‐generating and several caveats apply. First, the MR estimates show lifelong genetically determined differences in circulating metabolites and are not acute metabolic disturbances caused by the 77‐day treatment delay. Second, there were discordant directional estimates for the ApoB/ApoA1 ratio across the IEU and EBI databases; that is, it was positive in EBI but negative in IEU, and thus sensitive to instrument selection and population stratification. Due to this heterogeneity, this study did not include this ratio in the main causal inference. Thirdly, only one genome‐wide significant SNP was found in the 10 metabolic traits of the IEU panel, and Wald ratio estimation with inherent uncertainty had to be employed. Therefore, it cannot be concluded that MR has verified the treatment‐delay‐lipid‐PTC pathway.

Interestingly, a recent MR study on differentiated thyroid cancer (DTC) found that total cholesterol, HDL cholesterol and ApoB were causally associated with DTC risk, but the directionality and effect sizes were not the same as in our study (Ma et al. 2023). Given the different definitions of outcome (DTC vs. PTC), genetic instrument sets and population ancestry, such discrepancies were expected; thus, cross‐database triangulation needed to be employed rather than relying on a single GWAS source (Ma et al. 2023; Julkunen et al. 2023; Sanderson et al. 2021).

MR analysis explored the causal relationship between circulating metabolic biomarkers and did not consider the treatment delay. Although the inference that delayed treatment promotes the progression of PTC through alteration of lipid regulation has been supported by the convergence of transcriptomic and genetic data, further integration and prospective validation are required.

### 4.4 Clinical Implications: Towards Metabolically Informed Risk Stratification

The above results have good practical values for the preoperative management of PTC. At present, the reasons for selecting urgent surgery are mainly based on tumour histology, other diseases of the patient, resource availability, and lack biological information. The above data suggest that a delay in treatment may be associated with metabolic changes in the body that lead to VLDL‐rich and HDL‐poor blood. This condition was transcriptionally reflected in the tumour and directionally supported, but not confirmed by genetic analysis (He et al. 2022; Bian et al. 2021).

Thus, it is hoped that easily obtainable preoperative lipid data, such as VLDL‐triglyceride subfractions and HDL cholesterol, can be added to the risk classification system. The above indicators can help to identify patients who have suffered serious harm as a result of delayed treatment. Instead of using fixed time windows, we can also find individuals whose blood‐lipid profiles have been altered in a manner consistent with the cancer‐prone state suggested by our transcriptomic data. Therefore, early scheduling and simultaneous optimisation of metabolism (e.g., aiming for VLDL reduction via lifestyle modification or drugs) may be selected for this group (Kim et al. 2026).

However, this division system should be considered only as a hypothesis. The connection between the length of the delay and pre‐operative changes in lipids, whether these metabolic modifications can be reversed after starting treatment, and the reduction in oncologic effects of a longer delay all need to be verified prospectively. Our study offers the mechanistic reasons and genetic foundations for such trials, and puts forward the LPL‐APOC‐VLDLR axis as a potential biomarker panel and therapeutic target.

### 4.5 Deficiencies

Some methodological limitations affect the results of this study. First, the 77‐day threshold for defining a treatment delay was empirically determined from the cohort distribution and not based on an established clinical norm. Data‐driven cutoffs optimise the statistical separation but are prone to overfitting and have poor external generalisation; thus, prospective validation over fixed intervals is required.

Second, the clinical‐transcriptomic analysis used a retrospective, single‐center design and included 209 patients. The observed difference in CR rates for the short‐delay and long‐delay groups may be due to selection bias. However, the transcriptomic signals were confirmed in three independent GEO cohorts to address the above problems.

Third, the MR analysis assumed no horizontal pleiotropy and no sample overlap between the exposure and outcome GWAS. If the above assumptions are violated, especially weak instrument bias for metabolites with few instrumented SNPs, there may be a bias in the causal estimate. Heterogeneity testing and pleiotropy‐adjusted sensitivity analysis were conducted in this study to address the above deficiency, but residual confounding from unobserved genetic factors remains. The exploratory MR analysis found no significant exposure after correcting for multiple testing across 233 traits, and the nominal signals were not consistent in either outcome dataset. These results do not establish causality. Instrument strength was set by the selection threshold, as instruments were chosen at *p* < 1 × 10^−5^, every instrument exceeded the conventional *F* > 10 criterion, a minimum implied F of approximately 19.5, but per‐instrument *F*‐statistics were not individually reported, confounder screening for established PTC risk factors was not carried out, and outlier‐robust methods such as MR‐PRESSO were not used (Verbanck et al. 2018). Residual weak‐instrument bias for metabolites with few genome‐wide significant variants cannot be ruled out entirely.

Fourth, our outcome indicator was CR instead of disease‐free or overall survival. CR is clinically meaningful, but it does not fully reflect the long‐term recurrence characteristics. It has not been determined whether VLDL dysregulation prior to treatment offers extended survival benefits for some people.

Finally, the proposed LPL‐APOC‐VLDLR mechanistic axis was derived from observational and genetic data without experimental functional validation. Direct evidence that VLDL‐rich serum promotes the proliferation or migration of PTC cells by means of these particular receptors would offer strong support for the causal hypothesis.

## 5 Conclusion

In short, this study identified a treatment‐delay‐associated transcriptomic signature that indicates the activation of VLDL uptake machinery in PTC, and found that genetically predicted VLDL subfractions were directionally correlated with but did not robustly support a contribution to thyroid malignancy risk. Based on the above convergent observations, the preoperative waiting period has been viewed not as idle time but as a metabolically active state with oncological risks. The future risk‐stratification system will add pre‐operative lipidomic analysis, use readily obtainable VLDL and HDL indicators, and identify individuals at high risk of harm from treatment delays. The specific metabolic threshold at which urgent intervention is required and favourable strategies for metabolic optimisation during the waiting period need to be determined by prospective longitudinal studies.

## Author Contributions

**Yinfeng Li:** conceptualization, investigation, writing – original draft, writing – review and editing, visualization, validation, methodology, software, formal analysis, project administration, data curation, supervision, resources. **Ge Meng:** investigation, software, data curation. **Min Peng:** investigation, data curation, supervision, software. **Guangyan Chen:** methodology, software, formal analysis, resources. **Yin‐Ping Zhang:** conceptualization, investigation, writing – review and editing, methodology, validation, supervision, formal analysis, project administration.

## Funding

The authors have nothing to report.

## Ethics Statement

This study utilized publicly available, de‐identified datasets. No direct human participants or animal subjects were involved. Therefore, no additional ethical approval was required.

## Conflicts of Interest

The authors declare no conflicts of interest.

## Supporting information


**Table S1:** Minimal data set underlying the findings of this study, including the full differential gene expression results from the cross‐cohort transcriptomic analyses (TCGA‐THCA and three independent GEO cohorts) and the complete two‐sample Mendelian randomization outputs for papillary thyroid carcinoma.

## Data Availability

The minimal data set underlying the findings, including differential expression results and Mendelian randomization outputs, is provided in full as Table S1. The publicly available datasets used in this study are accessible via the following repositories: TCGA‐THCA transcriptomic and clinical data from the Genomic Data Commons Data Portal (https://portal.gdc.cancer.gov/); GEO datasets GSE29265, GSE33630 and GSE60542 from the NCBI Gene Expression Omnibus (https://www.ncbi.nlm.nih.gov/geo/); GWAS summary statistics for metabolic biomarkers from Karjalainen et al. (2024, Nature, https://doi.org/10.1038/s41586‐024‐07148‐y); and thyroid cancer GWAS data from the EBI OpenGWAS (ebi‐a‐GCST90018929) and IEU OpenGWAS (ieu‐a‐1082) databases (https://gwas.mrcieu.ac.uk/). All analytical pipelines were implemented in R (v4.2.2) using publicly available packages (TCGAbiolinks, limma, clusterProfiler, XGBoost, TwoSampleMR).
